# Polymorphisms in the A118G SNP of the OPRM1 gene produce different experiences of opioids: A human laboratory phenotype–genotype assessment

**DOI:** 10.1111/adb.13355

**Published:** 2023-12-19

**Authors:** Kelly E. Dunn, Andrew S. Huhn, Patrick H. Finan, Ami Mange, Cecilia L. Bergeria, Brion S. Maher, Jill A. Rabinowitz, Eric C. Strain, Denis Antoine

**Affiliations:** ^1^ Department of Psychiatry and Behavioral Sciences Johns Hopkins University School of Medicine Baltimore Maryland USA; ^2^ Department of Anesthesiology University of Virginia School of Medicine Charlottesville Virginia USA; ^3^ Yale School of Medicine New Haven Connecticut USA; ^4^ Department of Mental Health Johns Hopkins University School of Public Health Baltimore Maryland USA

**Keywords:** A118G, addiction, hydromorphone, opioid, OPRM1, phenotype, risk

## Abstract

Allelic variations in the A118G SNP of the OPRM1 gene change opioid signaling; however, evaluations of how allelic differences may influence opioid effects are lacking. This human laboratory paradigm examined whether the AA versus AG/GG genotypes determined opioid response profiles. Individuals with limited opioid exposure (N = 100) completed a five‐day within‐subject, double‐blind, placebo‐controlled, residential study. Participants were admitted (Day 1), received 4 mg hydromorphone (Day 2) and 0 mg, 2 mg and 8 mg hydromorphone in randomized order (Days 3–5) and completed self‐reported visual analog scale (VAS) ratings and Likert scales, observed VAS, and physiological responses at baseline and for 6.5 h post‐dose. Outcomes were analysed as peak/nadir effects over time as a function of genotype (available for N = 96 individuals; AG/GG = 13.5%, AA = 86.4%). Participants with AG/GG rated low and moderate doses of hydromorphone as significantly more positive (e.g., Good Effects VAS, coasting, drive, friendly, talkative, stimulation) with fewer negative effects (e.g., itchy skin, nausea, sleepiness), and were also observed as being more talkative and energetic relative to persons with AA. Persons with AG/GG were less physiologically reactive as determined by diastolic blood pressure and heart rate, but had more changes in core temperature compared with those with AA. Persons with AA also demonstrated more prototypic agonist effects across doses; persons with AG/GG showed limited response to 2 mg and 4 mg. Data suggest persons with AG/GG genotype experienced more pleasant and fewer unpleasant responses to hydromorphone relative to persons with AA. Future studies should replicate these laboratory findings in clinical populations to support a precision medicine approach to opioid prescribing.

## Graphical abstract

A human laboratory study conducted with individuals who had little to no prior exposure to opioids determined that the A118G SNP of the OPRM1 gene that codes for the mu opioid receptor changed the experience of opioids such that persons with the minor allele (AG/GG) had more pleasant and fewer adverse effects of several doses of hydromorphone relative to persons with the major (AA) allele.

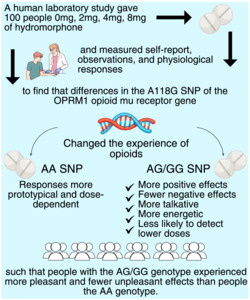

## 1 INTRODUCTION

Only 12% of the estimated >10 million persons in the United States who report lifetime use of an opioid develop opioid use disorder (OUD),^1^ suggesting only a subgroup of persons who are exposed to opioids develop problematic opioid use behaviours.^2^ To date, there have been limited evaluations of opioid response profiles; however, existing clinical reports^3^ and laboratory studies^4^, ^5^, ^6^, ^7^ suggest that some individuals demonstrate dose‐dependent responses to opioids whereas other individuals do not differentiate even high doses of opioids from placebo across a variety of patient‐reported, observed, and physiological outcomes (even when offered money for correctly differentiating between an opioid and a placebo).^5^, ^8^ These reports are primarily based upon persons who have a history of OUD, which is a population that has a demonstrated ability to interoceptively detect opioids. Much less is understood about the experience of opioids at the time of initiation, prior to development of acute or sustained tolerance. People who developed OUD have reported that their initial opioid exposure was uniquely differently, namely more pleasurable and stimulating in nature, than what was reported by persons who consumed opioids but did not develop OUD.^6^, ^9^, ^10^, ^11^ Features of this response profile are consistent with the empirically established low response behavioural phenotype for alcohol that is characterized by muted effects to alcohol and which reliably predicts future development of alcohol problems.^12^, ^13^, ^14^, ^15^, ^16^, ^17^ The low response phenotype for alcohol framework is valuable because it provides a measurable and observable pattern of responses that can be used to signal unique risk for future problematic use. The low response phenotype has never been examined in the context of opioids.

This study empirically examined differences in phenotypic response to multiple doses of an opioid that were administered in a laboratory setting to primarily opioid‐naïve persons. Data were further analysed in the context of polymorphic variations in the OPRM1 gene that codes for the mu opioid receptor. The specific target was the A118G functional polymorphism (rs1799971) that causes guanine (G) to be substituted for adenine (A) and triggers asparagine to replace aspartic acid. This ultimately reduces OPRM1‐related opioid signaling and could theoretically reduce opioid efficacy for individuals who have the minor allele (representing 4%–40% of the population).^18^, ^19^, ^20^, ^21^ Such a change could engender a low response profile. Notably, retrospective and correlative examinations of A118G in opioid response report that persons with the minor genotype (AG/GG) require higher opioid doses to achieve analgesia and adequate suppression of opioid withdrawal relative to persons who have the major genotype (AA),^22^, ^23^, ^24^, ^25^ further supporting this hypothesis.

The following analyses are based upon a within‐subject, double‐blind, randomized, placebo‐controlled, human laboratory study that examined whether individuals who had the AA or AG/GG versions of the A118G genotype experienced functional differences in response to low, medium and high doses of the prototypical mu agonist opioid hydromorphone. Data were collected from healthy individuals with no history of OUD, the majority of whom were opioid naïve. Phenotypic response profiles were assessed using an established Food and Drug Administration (FDA) drug assessment framework.^26^ The study hypothesized that persons with the AG/GG genotype (13.5% of participants) would exhibit lower sensitivity to opioids relative to those with the AA genotype (86.4% of participants), which may be indicative of a low response profile. Enrolling individuals who had limited or no prior experience with opioids eliminated the role that learned history from prior opioid exposure might have on responding and allowed modeling of opioid‐related risk among persons at the time of early opioid exposure.

## 2 METHODS

### 2.1 Participants

Healthy individuals (N = 100) were recruited via website postings to participate in a residential within‐subject study between 06/2015 and 02/2020. Eligible participants were required to be 21–50 years old, provide a urine sample that tested negative for amphetamines, barbiturates, benzodiazepines, cannabinoids, cocaine (benzoylecgonine), methamphetamine, opioids, oxycodone and phencyclidine, and be deemed medically eligible to take the study medication. Individuals who had chronic pain, active opioid prescriptions, met Diagnostic and Statistical Manual of Mental Disorders, Fifth Edition (DSM‐5) criteria for current or lifetime alcohol or substance use disorder (including opioids), reported any opioid use in the past 5 days or any illicit substance use in the past 7 days, had a body mass index (BMI) > 30, were pregnant or breastfeeding, had a known allergy to hydromorphone or other opioids, or had any clinically significant medical and/or psychiatric illness that was judged to possibly interfere with participation were excluded. This study was approved by the Johns Hopkins University School of Medicine Institutional Review Board (IRB) and registered as NCT02360371 (full protocol available). All participants provided voluntary informed consent to participate.

### 2.2 Eligibility screening

Patient‐reported measures and a semi‐structured clinical interview (MINI International Neuropsychiatric Interview^27^) were used to establish eligibility. Participants also provided blood and urine samples that were tested for medical eligibility, recent substance exposure, and pregnancy (as applicable), and completed a medical history, electrocardiogram (ECG) and physical examination prior to study admission.

### 2.3 Study design

Eligible participants were admitted to a Clinical Research Unit (CRU) for a 5‐day residential study on Day 1. Female participants were admitted during the follicular stage of their cycle to standardize hormonal effects on outcomes. Double‐blinded study drug (described below) was administered on Days 2–5. On drug administration days, participants received a standardized breakfast and completed baseline ratings of study measures before receiving study medication (approximately 08:30). After dosing, participants repeated ratings (described below) every 20 min for the first hour and every 30 min thereafter for a 6.5‐hour period (14 post‐drug timepoints total). Participants who smoked cigarettes (N = 11) were permitted to smoke during the session at standardized timepoints to minimize the acute effects of nicotine and nicotine withdrawal on ratings. Participants were discharged from the study and transported home at the end of Day 5 once acute drug effects had remitted.

### 2.4 Randomization

To minimize response bias, participants and research staff were all blinded to the drug under investigation and were informed participants might receive one or more of the following types of medications: benzodiazepines, opioids, stimulants, over‐the‐counter medications and/or placebo. The study medication was hydromorphone (0 mg, 2 mg, 4 mg, 8 mg), which was over‐encapsulated and administered in an oral formulation to all participants. All participants were scheduled to receive one administration of each dose (on separate days). Hydromorphone was selected as the prototypical opioid because its profile of effects is similar to heroin^28^ and it is not differentially metabolized by P450 enzymes.^29^ Study drug administration began on Day 2 and for safety reasons was always a nonrandomized dose of 4 mg. Participants who displayed elevated agonist effects in response to the 4 mg hydromorphone dose (i.e., vomiting) were housed overnight for observation and discharged from the study the following day once effects had remitted. For all other participants, the study pharmacist, who had no participant interaction, randomly assigned the order of remaining hydromorphone doses (0 mg, 2 mg and 8 mg) across study Days 3–5.

### 2.5 Drug effect ratings

#### 2.5.1 Participant ratings

Consistent with recommendations for assessing opioid agonist effects^30^, the following series of general visual analog scale (VAS) ratings (assessed on a 0 [not at all]–100 [extremely] point scale) were collected at each timepoint: Drug Effects, Good Effects, Bad Effects, High, and Like the Way I Feel. The following additional opioid‐specific VAS questions were also queried, representing pleasant (Elated, Energized, Stimulated, Talkative) and unpleasant (Difficulty Concentrating, Heavy Head, Nausea, Sedation, Sleepy, Sluggish) agonist effects. An assessment of pleasant (n = 11, carefree, coasting, drive, drunken, energetic, good mood, flushing, friendly, nodding, pleasant sick, relaxed) and unpleasant (n = 12; blurred vision, dry mouth, headache, feeling loose/limp, mentally slowed down, nervous, restless, shaky, sick to stomach, skin itchy, tense/jittery, turning of stomach) opioid agonist symptoms was also collected on a 0 (none at all)–4 (extremely) point Likert scale at each study timepoint.

#### 2.5.2 Observed VAS ratings

Blinded observers rated participant behaviour on general VAS (0–100) measures of Drug Effects, Good Effects, Bad Effects, and High, as well as specific VAS (0–100) pleasant (Energized, Talkative, Stimulated) and unpleasant (Difficulty Concentrating, Sedated, Sleepy, Inactive) effects, concurrent with patient‐reported ratings. Observers were trained on how to complete the ratings and, per protocol, all participants were rated by the same single observer during the duration of their participation to support within‐subject analyses.

#### 2.5.3 Physiological measures

Systolic and diastolic blood pressure (mm Hg), heart rate (beats per minute [bpm]), temperature (°F), oxygen saturation (%), respiration rate over 30 s (breaths per minute [brpm]) and pupil diameter (millimetre [mm]) as assessed using a pupilometer (Neuroptics, Irvine CA) were collected at each timepoint.

### 2.6 Genetic analyses

Whole blood was collected upon study admission (prior to study drug administration) and analysed using the Global Screening Array Genome‐Wide Association Screening (GWAS) chip by the Johns Hopkins University Genetic Core Laboratory.

### 2.7 Data analyses

A power analysis assuming a general linear model strategy to assess the effect of genotype on phenotype, unequal group sizes and a constant effect size difference between genotype groups (at each dose other than placebo) determined N = 100 would yield >80% power for modest effect sizes of approximately 0.45–0.67, consistent with other studies examining non‐opioid response differences in OPRM1.^31^ Independent group *t*‐tests (continuous) and chi‐squares (dichotomous) analyses compared demographic differences between persons with the AG/GG versus AA genotypes. Main effects of the A118G genotype were assessed by running three independent linear regression models fit with restricted maximum likelihood estimates that included a random intercept and autoregressive covariance structure. Outcomes for all analyses were independent VAS or Likert ratings and physiological outcomes. The first model assessed peak (patient and observed measures) or nadir (physiological) outcomes, representing the strongest drug effects that day, which were derived for each dose level and assessed for main effects of dose and A118G and dose × A118G interactions. A second model then examined raw ratings as a function of time during the session, assessing main effects of dose, A118G, time and all interactions. A third model examined area‐under‐the‐curve; since results from this model did not vary from the primary analyses, those outcomes are not reported here. All analyses were conducted using SAS version 9.4. Few data were missing so no corrections for missing data were made. Finally, because of the exploratory nature of this study, data were not corrected for multiple comparisons and alphas were set at 0.05 for all analyses.

## 3 RESULTS

### 3.1 Participants

One hundred (50 men [M], 50 women [F]) healthy individuals were enrolled into the study and received ≥1 dose of study drug (Table 1; Figure S1). Participants were, on average, 33.7 (SD = 9.1) years old, and Black/African American (46%), White/Caucasian (38%), Asian (5%) or identified as more than one race (6%). Eight percent identified as Hispanic, and only one participant endorsed past 30‐day exposure to opioids (Table 1). Fifteen participants (5M, 10F) showed elevated agonist effects after receiving 4 mg hydromorphone and were withdrawn from the study after demonstrating full remittance of effects by Day 3; one participant (F) withdrew on Day 4 prior to receiving study drug that day. Genotyping was unavailable for four participants. Of the remaining genotyped sample (n = 96), 13 (13.5%) participants were found to be AG/GG and 83 (86.5%) participants were found to be AA for A118G.

**TABLE 1:** Participant sample.

	Total	AA variant	AG/GG variant
	(N = 100)	(N = 83)	(N = 13)
Demographics			
Male (%, N)	49 (49)	47.0 (39)	69.2 (9)
Age (mean years, SD)	33.7 (9.1)	33.4 (8.9)	37.6 (10.0)
Race^a^ (%, N)			
American Indian/Pacific Islander	2.0 (2)	1.2 (1)	7.7 (1)
Asian	5.1 (5)	4.8 (4)	7.7 (1)
Black/African American	46.4 (46)	50.1 (42)	15.4 (2)
White/Caucasian	38.3 (38)	33.7 (28)	69.2 (9)
More than one race	6.1 (6)	7.2 (6)	0
Not reported	2.0 (2)	2.4 (2)	0
Hispanic ethnicity (%, N)	8.1 (8)	6.0 (5)	23.1 (3)
Body mass index (mean, SD)	24.7 (3.1)	24.6 (3.2)	26.1 (3.2)
Drug experience			
Opioids			
Past 30‐day use (%, N)	1 (1)	1 (1)	0
Past 30 days (mean days use, SD)	0.01 (0.1)	0.01 (0.1)	0
Lifetime (mean years use^b^, SD)	0.2 (1.5)	0.2 (1.5)	0.1 (0.28)
Alcohol			
Past 30‐day use (%, N)	54.5 (54)	56.6 (47)	46.2 (6)
Past 30 days (mean days use, SD)	1.7 (2.6)	1.7 (2.7)	2.0 (2.7)
Lifetime (mean years use^b^, SD)	9.1 (8.5)	9.5 (8.2)	8.6 (10.7)
Cannabis			
Past 30‐day use (%, N)	6.1 (6)	7.2 (6)	0
Past 30 days (mean days use, SD)	0.2 (1.3)	0.28 (1.4)	0
Lifetime (mean years use^b^, SD)	1.7 (3.6)	2.0 (3.8)	0.2 (0.4)
Tobacco			
Past 30‐day use (%, N)	10 (10)	9 (10.8)	1 (7.6)
Past 30 days (mean days use, SD)	5.8 (3.2)	6.2 (3.3)	10
Lifetime (mean years use^b^, SD)	8.4 (10.4)	6 (7.6)^c^	30^c^

*Note*: No additional between‐group differences met statistical significance.

^a^ Although race was not significant as a between‐group variable when all categories were examined independently, when collapsed into Black/African American, White/Caucasian, and Other categories, differences achieved significance (F[1,96] = 3.69, *p* = 0.029).

^b^ Mean lifetime years of use were operationalized as ‘number of weeks that use occurred at least 3 times weekly’ rounded into years and derived from Addiction Severity Index reports (opioids, alcohol, cannabis) or directly from participants (tobacco).

^c^ Significant between‐group difference (t (8)=2.99, *p* = 0.017).

### 3.2 Participant ratings

#### 3.2.1 VAS

**FIGURE 1:**
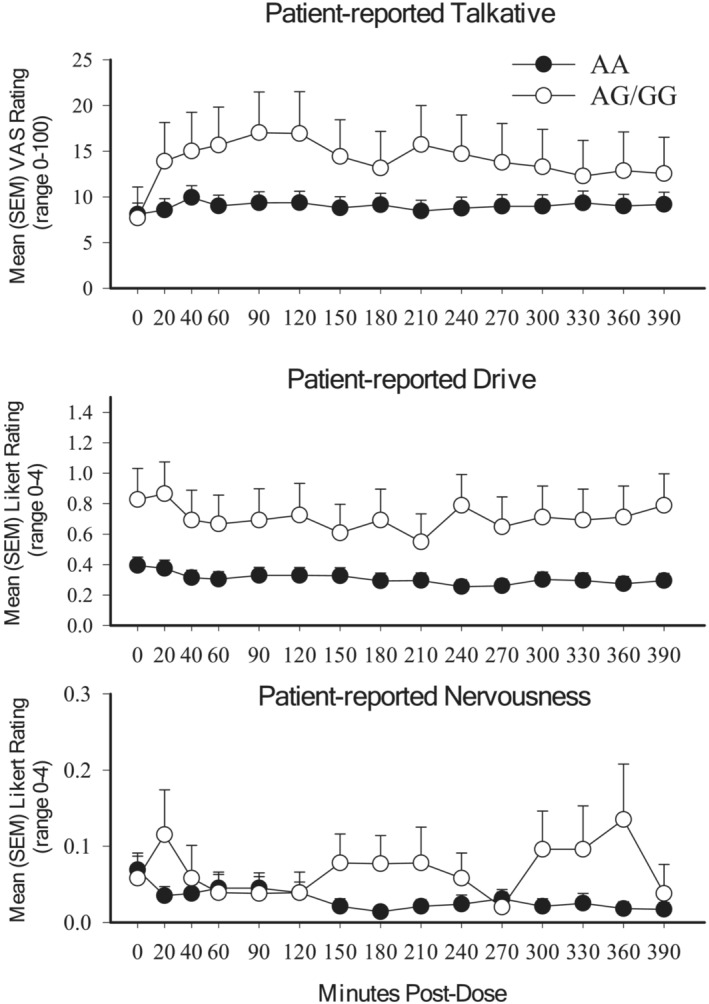
Significant gene‐based outcomes collapsed across dose. Data represent mean ratings (Y‐axis) collapsed across dose as a function of minutes post‐dose (x‐axis) for persons with the AA (closed) or AG/GG (open) version of the A118G SNP for outcomes related to patient‐reported talkativeness (F[14,1286] = 2.37, *p* = 0.003; top), drive (F[14,1286] = 2.12, *p* = 0.009; middle) and nervousness (F[14,1286] = 2.68, *p* = 0.001; bottom)

**FIGURE 2:**
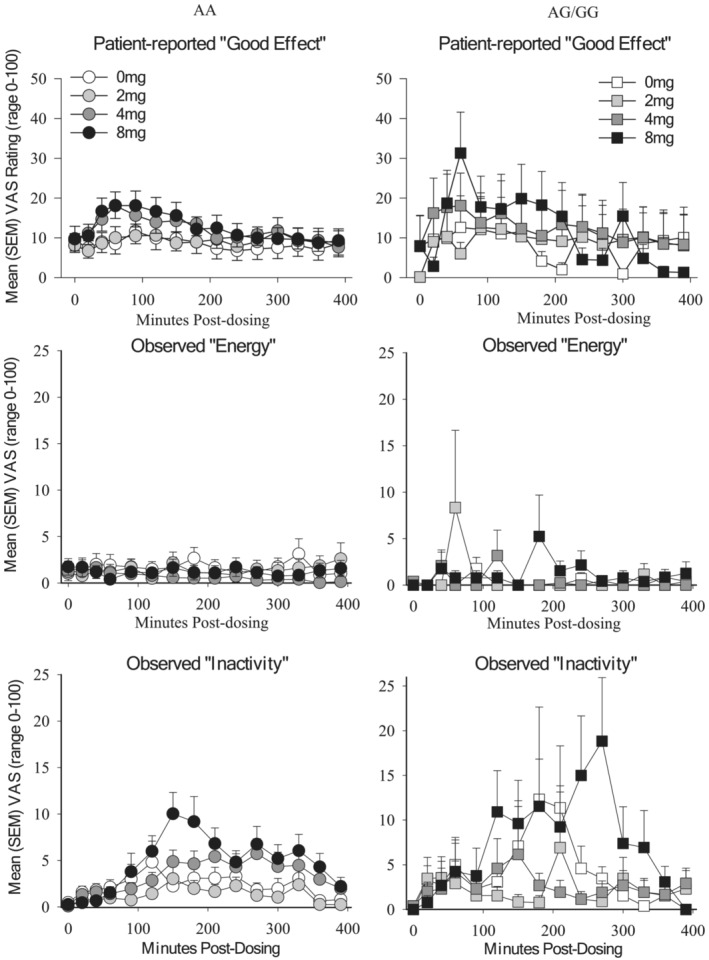
Significant gene‐based interactions. Mean ratings (Y‐axis) as a function of dose over minutes post‐dose (x‐axis) for persons with the AA (circle) and AG/GG (square) alleles of the A118G SNP for patient‐reported ratings of good effects (F[14,1286] = 2.8, *p* = 0.001; top panel), observed levels of energy (F[42,3245] = 1.7, *p* = 0.004; middle panel), and observed levels of inactivity (F[42,3245] = 1.6, *p* = 0.004, bottom panel)

Analysis of peak effects revealed main effects of dose on the majority of general and specific VAS ratings, with the exception of VAS Elated and Talkative (Table 2); no main effects of A118G or significant interactions were observed. Time‐based analyses also revealed several main effects of hydromorphone dose and time on general VAS ratings of Drug Effects, Bad Effects and High as well as the specific VAS ratings of Difficulty Concentrating, Heavy Head, Sedation, Sleepy, Slow Thoughts and Sluggish (Table 3). Significant main effects of gene revealed that individuals with the AG/GG genotype experienced greater Stimulation and lower Nausea and Sleepiness relative to individuals with the AA genotype, independent of dose (Table 3; Figure 1). A significant A118G × time × dose interaction was observed for VAS ratings of Good Effects (Figure 2) wherein individuals with the AG/GG genotype experienced a rapid escalation in Good Effects within 60 min of receiving 8 mg (but not 2 mg or 4 mg) hydromorphone, in contrast to individuals with the AA genotype who experienced a more prototypical dose‐dependent increase across doses (F[42,3319] = 1.6, *p* = 0.01). This rapid escalation was also observed for VAS general ratings of Drug Effects and High (Figure S2), but did not reach statistical significance (Table 3).

**TABLE 2:** Peak VAS and nadir physiological outcomes.

	A118G AA				A118G AG/GG					
	Hydromorphone dose				Hydromorphone dose				Main effects	
	0 mg	2 mg	4 mg	8 mg	0 mg	2 mg	4 mg	8 mg	A118G	Dose
Peak patient‐reported VAS ratings (0–100)										
Nonspecific ratings										
Drug effects	11.0 (20.0)	10.9 (20.6)	29.6 (29.0)	39.5 (32.1)	13.3 (21.3)	10.7 (16.4)	23.6 (27.0)	39.2 (37.3)	0.867	**<.0001**
Good effects	17.7 (29.5)	15.8 (29.3)	34.5 (38.0)	30.5 (33.3)	15.3 (29.3)	15.2 (27.8)	23.1 (30.9)	32.3 (37.5)	0.736	**0.000**
Bad effects	5.8 (15.0)	6.2 (16.4)	16.7 (24.9)	22.5 (28.2)	6.9 (13.6)	5.6 (14.4)	9.3 (11.8)	17.5 (26.6)	0.519	**0.000**
High	6.6 (16.3)	5.5 (15.3)	19.1 (27.1)	22.4 (27.2)	7.2 (13.2)	4.4 (5.8)	12.5 (19.7)	25.4 (29.6)	0.854	**<.0001**
Like the Way I Feel	46.9 (39.8)	51.1 (40.2)	63.7 (35.7)	52.9 (36.6)	44.5 (38.3)	48.9 (40.0)	58.9 (36.2)	60.9 (33.1)	0.991	**0.001**
Pleasant ratings										
Elated	8.6 (20.8)	9.7 (23.7)	14.0 (25.9)	10.1 (20.5)	13.3 (29.7)	16.6 (28.7)	15.7 (30.1)	16.9 (33.4)	0.439	0.382
Energized	12.4 (25.1)	16.9 (29.8)	25.3 (31.7)	15.5 (27.5)	16.9 (32.2)	21.3 (37.5)	25.9 (38.3)	19.1 (35.7)	0.662	**0.004**
Stimulated	7.6 (20.8)	7.9 (20.8)	16.3 (28.1)	10.8 (22.8)	15.0 (30.2)	13.6 (29.1)	19.5 (31.0)	16.1 (32.1)	0.399	**0.031**
Talkative	13.5 (25.7)	14.9 (28.1)	20.6 (30.7)	16.7 (25.8)	16.3 (32.2)	23.1 (39.0)	24.6 (36.9)	21.2 (32.8)	0.521	*0.056*
Unpleasant ratings										
Difficulty concentrating	5.6 (14.4)	3.9 (7.7)	15.0 (22.1)	16.7 (22.8)	6.5 (11.0)	6.5 (12.9)	9.6 (16.8)	22.9 (28.1)	0.783	**<.0001**
Heavy head	5.6 (13.5)	4.3 (9.7)	14.3 (23.1)	14.7 (20.0)	3.3 (5.7)	2.6 (4.0)	5.6 (8.5)	16.4 (26.7)	0.459	**0.000**
Nausea	4.0 (13.4)	5.0 (15.3)	12.4 (20.8)	16.5 (26.7)	2.7 (3.6)	1.2 (2.8)	7.1 (13.9)	11.6 (15.3)	0.346	**0.001**
Sedation	6.4 (16.2)	3.9 (9.9)	15.3 (24.3)	12.6 (23.0)	7.3 (11.6)	5.3 (16.4)	6.3 (8.6)	18.3 (26.1)	0.972	**0.002**
Sleepy	19.1 (28.3)	17.1 (24.8)	35.3 (31.3)	32.0 (32.2)	14.3 (20.1)	9.3 (11.4)	12.8 (20.3)	26.8 (31.6)	0.165	**0.000**
Slow thoughts	5.4 (14.1)	3.8 (11.0)	13.9 (22.0)	15.0 (20.5)	6.3 (12.3)	4.0 (11.2)	7.6 (10.0)	22.5 (28.1)	0.879	**<.0001**
Sluggish	12.1 (23.0)	9.6 (16.5)	24.8 (28.1)	26.7 (28.1)	9.8 (15.8)	5.3 (7.6)	9.6 (11.4)	25.8 (29.2)	0.318	**<.0001**
Nadir physiological outcomes										
Systolic blood pressure	109.3 (9.6)	109.6 (9.4)	107.6 (10.7)	110.0 (9.1)	109.6 (5.9)	111.3 (8.5)	109.1 (13.7)	107.8 (5.7)	0.913	0.350
Diastolic blood pressure	63.5 (7.3)	62.9 (6.7)	62.0 (6.6)	64.2 (6.9)	62.3 (6.3)	64.9 (8.1)	63.6 (8.3)	63.2 (7.7)	0.816	0.603
Pulse (bpm)	62.4 (9.3)	61.2 (7.8)	59.2 (7.9)	59.5 (8.7)	64.7 (9.6)	62.6 (9.5)	61.5 (9.1)	61.6 (10.9)	0.372	**0.001**
Pupil diameter (mm)	3.5 (0.7)	3.2 (0.6)	3.1 (0.6)	2.7 (0.5)	3.4 (0.7)	3.2 (0.7)	3.1 (0.4)	2.8 (0.5)	0.908	**<.0001**
Respiration (30 s)	6.6 (0.7)	6.7 (0.7)	6.8 (0.9)	6.6 (0.9)	7.1 (0.8)	6.8 (0.8)	6.3 (0.6)	6.4 (0.6)	0.930	**0.005**
Oxygen saturation	97.7 (1.2)	97.7 (1.2)	97.7 (1.0)	97.4 (1.3)	97.0 (1.7)	97.6 (0.8)	97.2 (0.8)	97.1 (1.3)	0.137	0.220
Temperature (F)	97.6 (0.5)	97.7 (0.3)	97.4 (0.4)	97.4 (0.6)	97.3 (1.2)	97.5 (0.3)	96.4 (2.2)	97.1 (0.9)	**0.003**	**<.0001**

*Note*: Values represent mean (standard deviation) unless otherwise noted. Italicized and bolded values reflect outcomes that trended or met significance, respectively.

Abbreviations: bpm, beats per minute; mg, milligram: mm, millimetre: VAS, visual analog scale.

**TABLE 3:** Time‐based analyses.

	A118G AA				A118G AG/GG						
	Hydromorphone dose				Hydromorphone dose				Main effects		
	0 mg	2 mg	4 mg	8 mg	0 mg	2 mg	4 mg	8 mg	A118G	Dose	Time
Patient‐reported VAS Ratings (0–100)											
Nonspecific ratings											
Drug effects	3.4 (11.03)	2.22 (7.56)	11.38 (20.36)	15.39 (23.49)	3 (9.22)	2.59 (7.71)	7.72 (13.86)	15.61 (26.11)	0.527	**<.0001**	**<.0001**
Good effects	8.5 (22.84)	9.17 (24.21)	12.01 (24.78)	12.53 (26.05)	8.87 (24.61)	8.97 (25.21)	12.36 (27.73)	12.08 (25.2)	0.872	0.709	**0.001**
Bad effects	1.73 (7.45)	1.22 (5.37)	6.38 (15.99)	8.62 (17.89)	1.68 (7.27)	1.46 (5.91)	2.04 (4.71)	7.11 (18.93)	0.193	**0.000**	**0.002**
High	2.01 (7.69)	1.73 (7.24)	5.3 (14.3)	6.89 (15.8)	1.74 (6.01)	0.76 (2.24)	2.87 (8.08)	7.07 (15.4)	0.460	**0.001**	**<.0001**
Like the Way I Feel	35.5 (37.23)	37.75 (39.08)	32.91 (35.7)	29.62 (34.8)	33.92 (36.97)	35.27 (37.36)	38.65 (37.18)	38.81 (35.49)	0.436	0.945	0.281
Pleasant ratings											
Elated	4.8 (17.14)	5.31 (17.67)	4.96 (16.1)	4.47 (14.93)	10.22 (27.45)	8.65 (26.09)	9.84 (27.31)	12.29 (28.89)	0.068	0.981	0.514
Energized	6.98 (20.77)	8.49 (23.02)	10.34 (23.44)	6.63 (19.46)	9.87 (26.61)	10.54 (27.44)	13.01 (28.99)	13.5 (29.6)	0.230	0.806	0.090
Stimulated	3.91 (14.84)	4.72 (17.28)	6.15 (18.47)	3.96 (14.57)	9.64 (27.25)	8.8 (26.1)	10.34 (26.76)	12.05 (28.85)	**0.038**	0.933	**0.000**
Talkative	8.66 (21.33)	9.37 (22.5)	9.1 (20.15)	8.83 (20.61)	12.14 (27.72)	14.02 (31.05)	15.11 (30.16)	14.44 (29.6)	0.170	0.933	**0.001**
Unpleasant ratings											
Difficulty concentrating	1.84 (6.24)	1.19 (4.07)	5.34 (14.39)	5.39 (12.53)	1.22 (4.24)	1.9 (5.88)	2.52 (7.97)	7.31 (18.69)	0.773	**0.003**	**<.0001**
Heavy head	2.03 (7.06)	1.42 (5.49)	5.03 (13.93)	5.01 (12.95)	0.82 (2.56)	1.04 (2.62)	1.87 (4.47)	6.01 (17.88)	0.399	**0.019**	**0.009**
Nausea	1.53 (7.95)	1.35 (5.67)	4.61 (12.75)	5.95 (15.72)	0.64 (1.94)	0.4 (1.26)	1.08 (4.32)	2.31 (6.46)	**0.031**	0.058	0.448
Sedation	2.25 (8.02)	1.21 (5.13)	5.15 (14.73)	5.12 (13.96)	1.68 (5.24)	2.07 (7.59)	1.79 (4.49)	6.69 (19.68)	0.698	**0.027**	**0.005**
Sleepy	6.09 (16.75)	5.52 (14.2)	14.48 (23.7)	13.98 (22.83)	4.76 (10.89)	2.96 (6.58)	4.23 (9.38)	11.01 (22.81)	**0.030**	**0.006**	**0.005**
Slow thoughts	1.99 (7.16)	1.42 (5.79)	5.28 (14.14)	5.03 (12)	1.68 (5.52)	1.66 (5.3)	2.26 (5.17)	8.14 (20.34)	0.850	**0.003**	**<.0001**
Sluggish	4.26 (12.78)	3.02 (8.71)	11.0 (21.78)	11.09 (19.26)	3.23 (8.35)	1.6 (3.74)	2.37 (5.31)	10.35 (21.49)	0.098	**0.002**	**<.0001**
Physiological outcomes											
Systolic blood pressure	122.28 (13.49)	122.9 (13.31)	119.82 (12.95)	122.58 (12.94)	122.9 (13.86)	123.03 (10.86)	123.23 (12.24)	122.14 (9.78)	0.270	0.636	0.621
Diastolic blood pressure	73.42 (8.74)	74.04 (9.43)	71.42 (9.27)	74.11 (9.73)	75.26 (9.87)	74.91 (10.13)	74.93 (10.25)	73.66 (8.9)	**0.024**	0.435	0.056
Pulse (bpm)	72.09 (12.82)	71.21 (12.43)	67.67 (11.2)	69.95 (13.03)	75.04 (13.29)	72.27 (11.5)	71.1 (11.66)	71.88 (11.25)	**0.030**	**0.034**	**<.0001**
Pupil diameter (mm)	4.2 (1.83)	3.86 (1.95)	3.73 (1.36)	3.34 (0.99)	4.06 (0.94)	3.95 (2.14)	3.85 (2.12)	3.42 (0.79)	0.654	**<.0001**	**0.001**
Respiration (30 s)	8.35 (7.48)	8.47 (8.1)	8.25 (6.4)	8.07 (5.93)	8.26 (2.93)	7.84 (1.07)	8.12 (6.59)	7.69 (1.01)	0.271	0.739	0.898
Oxygen saturation	97.86 (8.74)	97.78 (9.4)	98.19 (7.19)	97.96 (7.88)	97.97 (7.04)	98.59 (0.61)	97.92 (6.89)	98.44 (0.85)	0.391	0.923	0.905
Temperature (F)	97.55 (8.03)	97.43 (8.5)	97.59 (6.35)	97.48 (6.96)	98.11 (0.52)	98.03 (0.34)	97.41 (6.51)	97.87 (0.51)	0.259	0.890	0.964

*Note*: Values represent mean (standard deviation) unless otherwise noted. Italicized and bolded values reflect outcomes that trended or met significance, respectively.

Abbreviations: bpm, beats per minute; mg, milligram; mm, millimetre; VAS, visual analog scale.

#### 3.2.2 Likert ratings

Analyses of peak effects revealed significant main effects of dose on pleasant ratings (i.e., coasting, drive, drunken, energetic, flushing, friendliness, pleasant sick) and unpleasant ratings (i.e., blurred vision, feeling limp, mentally slowed down, sick to stomach, turning of stomach) (Table S1). A significant main effect of gene was observed for peak coasting (F[1,82] = 11.8, *p* = 0.001), whereby individuals with the AG/GG genotype experienced higher levels of coasting than individuals with the AA genotype independent of dose. No gene × dose interactions were observed for any peak Likert ratings.

Time‐based analyses revealed individuals with the AG/GG genotype experienced significantly higher levels of pleasant effects (i.e., coasting, drive, feeling friendly, feeling energetic, and stimulation), and lower levels of unpleasant effects (i.e., itchy skin, feeling sick to their stomach, nausea, sleepiness, and turning of stomach) relative to individuals with the AA genotype (Table S2). Significant A118G × time interactions revealed individuals with the AG/GG genotype experienced a more rapid escalation of talkativeness (F[14,1286] = 2.37, *p* = 0.003) and drive (F[14,1286] = 2.12, *p* = 0.009), and a more delayed onset of nervousness (F[14,1286] = 2.68, *p* = 0.001), compared with individuals with AA, independent of dose (Figure 1). A significant A118G × dose × time interaction was also evident for flushing (F[42,3320] = 1.41, *p* = 0.042) whereby individuals with AA experienced dose‐dependent increases in flushing but individuals with AG/GG group only experienced flushing at the 8 mg dose (Figure S2).

### 3.3 Observed ratings

Analysis of observed peak effects revealed significant main effects of dose on general observed effects (Drug Effects, Good Effects, Bad Effects, and High), and the specific rating of Inactivity (Table S1). No main effects of A118G or interactions on observed peak effects were identified. The time‐based analyses also identified significant main effects of dose on observed levels of general Drug Effects, High and Bad Effects, as well as specific effects including observed Sleepiness, and Inactivity (Table S2). A significant main effect of A118G on observed Talkativeness (F[1,95] = 29.1, *p* < 0.001) revealed that persons with the AG/GG variant were rated as being more talkative than persons with the AA variant, independent of dose. Two significant A118G × time × dose interactions were also identified. First, persons with the AG/GG variant were rated as displaying more energy in response to all hydromorphone doses relative to persons with the AA variant, and the time of peak energy ratings varied as a function of dose within individuals who had the AG/GG but not AA genotype (F[42,3245] = 1.69, *p* = 0.004; Figure 2). Second, persons with the AG/GG variant displayed higher but more delayed onset of inactivity relative to persons with the AA variant (F[42,3245] = 1.67, *p* = 0.004; Figure 2).

### 3.4 Physiological measures

In general, acute opioid effects in non‐physically dependent persons reduce physiologic ratings, so nadir outcomes were examined for these measures. Significant main effects of dose were identified for heart rate, temperature, respiration rate and pupil size (Table 2). A significant main effect of A118G was found for nadir temperature (F[1,82] = 9.7, *p* = 0.003), whereby persons with the AG/GG genotype demonstrated greater decreases in core temperature relative to persons with the AA genotype at the 4 mg dose. Time‐based analyses revealed significant main effects of dose on nadir heart rate and pupil diameter (Table 3). Significant main effects of A118G were also evident for nadir heart rate (F[1,95] = 4.8, *p* = 0.030) and diastolic blood pressure (F[1,95] = 5.2, *p* = 0.024), both of which revealed that individuals with the AG/GG genotype had higher values (i.e., were less sensitive to hydromorphone effects) on these outcomes compared with individuals with the AA genotype. No significant interactions were observed for physiological ratings.

## 4 DISCUSSION

Data from this human laboratory study provide evidence that some participants exhibit reduced response to opioids (possibly signaling a low response profile) and that these effects were clustered in persons who had the minor genotype (AG/GG) on the A118G SNP on the OPRM1 gene. Among persons with the AG/GG genotype, the 4 mg moderate dose of the prototypic opioid hydromorphone produced a profile of effects that is consistent with administration of a low dose of opioids. Specifically, this was characterized by higher self‐reported ratings on positive measures such as coasting, drive, energy, friendliness, stimulation and talkativeness, and lower ratings on unpleasant measures such as itchy skin, feeling sick to their stomach and nausea. Persons with the AG/GG genotype also demonstrated less prominent physiological responses to hydromorphone, evidenced by smaller reductions in heart rate and diastolic blood pressure, relative to persons with the AA genotype, and did not show the expected elevation in agonist effects until the 8 mg dose was administered. In contrast, persons with the AA genotype demonstrated more conventional dose‐dependent increases in responding across doses. Moreover, genotype was also associated with a different time course for some of the outcomes collected, suggesting both magnitude and speed of onset of opioid effects may distinguish the AA and AG/GG groups.

Blinded observers corroborated these outcomes by rating persons with the AG/GG genotype as being more talkative and exhibiting more energy compared with persons with the AA genotype, two outcomes commonly reported following exposure to low doses of opioids.^6^ Increased talkativeness is particularly noteworthy because it was reported by both participants and observers and has been empirically associated with finding a drug more reinforcing.^32^, ^33^ These collective symptom profiles suggest that persons with the AG/GG genotype may have been experiencing a lower sensitivity to opioids, which manifested as the lower opioid doses conferring more stimulating effects with fewer negative side effects than what was experienced by persons with the AA genotype. It seems possible that reduced opioid signaling resultant from the A118G polymorphisms could change mu‐receptor binding potential and underlie these different effect profiles,^19^ though this remains an empirical question.

These results are consistent with other studies that have reported differences in OPRM1 genotype confer functional changes in opioid response. For instance, the minor G allele has been found in in vitro studies to be associated with stronger binding for endogenous but not exogenous opioid agonists^34^, ^35^ and in a positron emission study to reduce mu receptor function.^36^ Variations of the OPRM1 genotype have also been associated with differences in opioid withdrawal severity^37^ and response to OUD pharmacotherapies,^38^, ^39^, ^40^, ^41^ further suggesting that allelic differences in the OPRM1 gene directly modify opioid efficacy and function. The data from this study now add to this knowledge base by suggesting that differences in the A118G SNP of the OPRM1 may influence opioid experience at the time of opioid initiation. Importantly, despite not reaching full statistical significance, the minor allele was present in more White/Caucasian participants than in participants of other racial and ethnic origins and in marginally more biological men than women. These data are consistent with general epidemiological sampling and support subsequent examinations of the effects observed here within these important demographic domains.

Notable limitations of this study include the fact that observed ratings were conducted by a single staff member for each participant and preclude determination of interrater reliability. This study also had a relatively small sample for a genotype‐based evaluation (which is somewhat offset by the extremely sensitive phenotyping procedure)^42^ and low representation of the G variant, which may have led to some results being underpowered. Nevertheless, these data can serve as initial evidence that the minor genotype (AG/GG) of the A118G SNP may reduce individual sensitivity to opioids relative to the major genotype (AA). Results are consistent with the low response phenotype that is well established as a risk phenotype in the context of alcohol use disorder, though the current analyses are not a definitive examination of this phenotype for opioids. Additionally, prior research has found that even highly phenotyped laboratory examinations of candidate gene effects may not be reliably replicated,^43^ raising concerns regarding the ultimate clinical utility of these findings.

## 5 CONCLUSIONS

These data provide initial evidence that the minor (AG/GG) genotype of the A118G SNP was associated with a reduced sensitivity to opioids relative to the major genotype (AA), a finding that is consistent with the low response phenotype that is well‐established as a risk phenotype in the context of alcohol use disorder but has not yet been examined in the context of opioids. Though results should be considered preliminary because of the low representation of the AG/GG genotype, these findings may elucidate a potentially important and testable behavioural phenotype for opioid risk (namely the evaluation of initial sensitivity as a potential metric for predicting future response profiles).

## AUTHOR CONTRIBUTIONS

Authors KED, PFH, BSM, ECS, and DA conceptualized the study; KED, ASH, PFH, AM, CLB, ECS, and DA managed study operations and enrollment, authors KED, ASH, BSM, JAR conducted statistical analyses, and all authors contributed to manuscript preparation.

## CONFLICT OF INTEREST STATEMENT

No authors have direct competing interests to report. In the past 3 years, KED has consulted or served as an advisor to study protocols with companies Mind Med, Inc. DemeRx, Cessation Therapeutics, and Indivior and has received funding through her university from the National Institute on Drug Abuse and Cure Addiction Now. ASH has consulted for Gilgamesh and received research funding from Indivior through his University. PHF has served on an advisory board for Ninnion Therapeutics. CLB has received research funding through her university from Canopy Growth Corporation and Pear Therapeutics. ECS has served on advisory boards and received grant funding or supplies for studies from, and/or consulted with: Ashley Addiction Treatment, Cerevel Therapeutics, Clearmind Medicine, Fast‐Track Drugs & Biologics, Masimo Corporation, UpToDate, Otsuka Pharmaceutical Development and Commercialization, and Pear Therapeutics.

## ETHICS APPROVAL STATEMENT

This study was approved by the Johns Hopkins School of Medicine Institutional Review Board.

## PATIENT CONSENT STATEMENT

Participants provided voluntary informed consent to participate.

## CLINICAL TRIAL REGISTRATION

NCT02360371.

## Supporting information


**Figure S1.** CONSORT Diagram.
**Figure S2.** Clinically‐interesting gene‐based outcomes: mean ratings (Y‐axis) as a function of gene × dose × minutes post‐dose (x‐axis) for persons with the AA (circle) and AG/GG (square) alleles of the A118G SNP for patient‐reported ratings of Drug Effects (F[42,3319] = 0.95, *p* = 0.56; top panel), High (F[42,3319] = 0.78, *p* = 0.84; middle panel) and Flushing (F[42,3320] = 1.41, *p* = 0.042; bottom panel).
**Table S1.** Peak Likert and Observed Outcomes.
**Table S2.** Likert and Observed Time‐based Analyses.

## Data Availability

Data can be made available via data sharing agreements by request.
