# Supplementary material for: Polymorphisms in the A118G SNP of the OPRM1 gene produce different experiences of opioids: A human laboratory phenotype–genotype assessment
Source: Addict Biol. 2023 Dec 19;29(1):e13355. doi: 10.1111/adb.13355 (PMC10898793; doi:10.1111/adb.13355)
Supplement: Supplementary file 1 — Figure S1. CONSORT Diagram. Figure S2. Clinically‐interesting gene‐based outcomes: mean ratings (Y‐axis) as a function of gene × dose × minutes post‐dose (x‐axis) for persons with the AA (circle) and AG/GG (square) alleles of the A118G SNP for patient‐reported ratings of Drug Effects (F[42,3319] = 0.95, p = 0.56; top panel), High (F[42,3319] = 0.78, p = 0.84; middle panel) and Flushing (F[42,3320] = 1.41, p = 0.042; bottom panel). Table S1. Peak Likert and Observed Outcomes. Table S2. Likert and Observed Time‐based Analyses. [file ADB-29-e13355-s001.docx]

Supplemental Figure 1. CONSORT Diagram

**Supplemental Figure 2.** **Clinically-interesting Gene-based Outcomes**

| Supplementary Table 1. Peak Likert and Observed Outcomes | | | | | | |  |  |  |  |  |  |
| --- | --- | --- | --- | --- | --- | --- | --- | --- | --- | --- | --- | --- |
|  |  |  | A118G AA | | | | A118G AG/GG | | | |  |  |
|  |  |  | Hydromorphone Dose | | | | Hydromorphone Dose | | | | Main Effects | |
|  |  |  | 0mg | 2mg | 4mg | 8mg | 0mg | 2mg | 4mg | 8mg | A118G | Dose |
| Peak Patient-reported Likert Ratings (0-4) | | |  |  |  |  |  |  |  |  |  |  |
|  | Pleasant Ratings | |  |  |  |  |  |  |  |  |  |  |
|  |  | Coasting | 0.1 (0.3 ) | 0 (0 ) | 0.3 (0.7 ) | 0.2 (0.5 ) | 0.6 (1.3 ) | 0.6 (1.2 ) | 0.7 (1.3 ) | 1.0 (1.3 ) | **0.001** | **0.000** |
|  |  | Drive | 0.4 (1.0 ) | 0.4 (1.0 ) | 0.7 (1.2 ) | 0.4 (0.9 ) | 1.0 (1.5 ) | 0.8 (1.5 ) | 1.4 (1.5 ) | 1.2 (1.6 ) | 0.078 | **<.0001** |
|  |  | Drunken | 0 (0 ) | 0 (0 ) | 0.1 (0.4 ) | 0.3 (0.7 ) | 0.1 (0.5 ) | 0 (0 ) | 0 (0 ) | 0.4 (0.9 ) | 0.951 | **<.0001** |
|  |  | Energetic | 1.2 (1.3 ) | 1.1 (1.3 ) | 1.5 (1.3 ) | 1.0 (1.3 ) | 1.3 (1.6 ) | 1.6 (1.7 ) | 2.0 (1.5 ) | 1.6 (1.9 ) | 0.230 | **0.026** |
|  |  | Flushing | 0 (0 ) | 0 (0 ) | 0.1 (0.3 ) | 0.2 (0.7 ) | 0 (0 ) | 0 (0 ) | 0.2 (0.5 ) | 0.3 (0.9 ) | 0.734 | **0.001** |
|  |  | Friendly | 1.7 (1.4 ) | 1.9 (1.4 ) | 2.1 (1.3 ) | 1.9 (1.4 ) | 1.9 (1.4 ) | 2.2 (1.6 ) | 2.5 (1.3 ) | 2.3 (1.6 ) | 0.428 | **0.001** |
|  |  | Pleasant Sick | 0 (0 ) | 0 (0 ) | 0.1 (0.4 ) | 0.1 (0.3 ) | 0 (0 ) | 0 (0 ) | 0 (0 ) | 0.3 (0.8 ) | 0.985 | **0.007** |
|  | Unpleasant Ratings | |  |  |  |  |  |  |  |  |  |  |
|  |  | Blurred Vision | 0 (0 ) | 0 (0 ) | 0 (0 ) | 0.1 (0.5 ) | 0 (0 ) | 0 (0 ) | 0 (0 ) | 0.1 (0.3 ) | 0.922 | **0.015** |
|  |  | Feel Limp or Loose | 0.1 (0.4 ) | 0 (0 ) | 0.3 (0.7 ) | 0.5 (1.0 ) | 0 (0 ) | 0 (0 ) | 0 (0 ) | 0.4 (0.9 ) | 0.488 | **<.0001** |
|  |  | Headache | 0.2 (0.5 ) | 0.2 (0.6 ) | 0.4 (0.8 ) | 0.4 (0.8 ) | 0.3 (0.7 ) | 0.2 (0.4 ) | 0.6 (0.9 ) | 0.4 (0.5 ) | 0.719 | 0.096 |
|  |  | Mentally Slowed Down | 0.2 (0.8 ) | 0.1 (0.6 ) | 0.8 (1.1 ) | 0.7 (0.9 ) | 0.4 (0.7 ) | 0.3 (0.8 ) | 0.3 (0.6 ) | 0.9 (1.0 ) | 0.975 | **0.000** |
|  |  | Nervous | 0.1 (0.5 ) | 0 (0 ) | 0.1 (0.5 ) | 0.1 (0.4 ) | 0.1 (0.5 ) | 0 (0.2 ) | 0.1 (0.5 ) | 0.3 (0.8 ) | 0.724 | 0.126 |
|  |  | Sick to Stomach | 0 (0 ) | 0 (0 ) | 0.4 (0.9 ) | 0.5 (0.9 ) | 0 (0 ) | 0 (0 ) | 0.1 (0.3 ) | 0.2 (0.5 ) | 0.120 | **0.017** |
|  |  | Skin Itchy | 0.1 (0.3 ) | 0.1 (0.3 ) | 0.2 (0.5 ) | 0.5 (0.9 ) | 0.3 (1.1 ) | 0.3 (0.8 ) | 0 (0 ) | 0.3 (0.6 ) | 0.852 | 0.202 |
|  |  | Turning of Stomach | 0.2 (0.6 ) | 0.2 (0.5 ) | 0.3 (0.6 ) | 0.6 (0.9 ) | 0 (0 ) | 0 (0 ) | 0.1 (0.3 ) | 0.3 (0.6 ) | 0.065 | **0.016** |
| Peak Observed VAS Ratings (0-100) | | |  |  |  |  |  |  |  |  |  |  |
|  | Nonspecific Ratings | |  |  |  |  |  |  |  |  |  |  |
|  |  | Drug Effects | 9.6 (18.7) | 6.1 (11.2) | 19.5 (21.9) | 25.0 (24.3) | 7.4 (11.6) | 4.7 (9.7) | 19.3 (21.4) | 22.9 (25.6) | 0.725 | **<.0001** |
|  |  | Good Effects | 2.4 (9.4) | 1.5 (6.7) | 3.9 (10.5) | 5.8 (15.0) | 1.4 (5.2) | 0 (0) | 0.8 (2.1) | 10.3 (22.3) | 0.893 | **0.001** |
|  |  | Bad Effects | 4.5 (14.1) | 3.3 (8.0) | 8.4 (15.6) | 13.2 (19.1) | 3.0 (8.5) | 0 (0) | 2.6 (5.6) | 6.1 (13.2) | 0.117 | **0.018** |
|  |  | High | 1.7 ( 8.1) | 0.9 (2.9) | 5.8 (13.1) | 8.9 (18.5) | 0 (0) | 0 (0) | 3.4 (8.7) | 9.8 (22.1) | 0.679 | **0.000** |
|  | Pleasant Ratings | |  |  |  |  |  |  |  |  |  |  |
|  |  | Energized | 4.7 (13.3) | 3.8 (14.3) | 3.7 (7.2) | 5.6 (14.6) | 3.2 (5.1) | 8.8 (27.7) | 3.6 (9.0) | 6.3 (16.2) | 0.735 | 0.506 |
|  |  | Talkative | 15.3 (17.3) | 17.3 (20.6) | 16.4 (20.5) | 19.8 (22.7) | 29.6 (25.3) | 24.6 (29.2) | 21.5 (23.4) | 30.3 (29.3) | *0.092* | 0.173 |
|  |  | Stimulated | 4.3 (12.9) | 2.9 (9.3) | 3.4 (7.9) | 5.3 (13.6) | 2.6 (6.8) | 9.6 (27.4) | 4.9 (9.1) | 6.1 (12.4) | 0.527 | 0.374 |
|  | Unpleasant Ratings | |  |  |  |  |  |  |  |  |  |  |
|  |  | Sedated | 3.5 (14.4) | 2.7 (7.6) | 6.6 (13.8) | 7.2 (17.1) | 3.8 (9.6) | 2.0 (5.0) | 5.1 (9.5) | 6.6 (13.1) | 0.841 | 0.154 |
|  |  | Sleepy | 12.7 (22.2) | 13.7 (20.2) | 26.0 (28.4) | 27.3 (28.5) | 19.8 (24.2) | 18.6 (27.4) | 11.8 (18.9) | 25.1 (24.8) | 0.838 | *0.077* |
|  |  | Difficulty Concentrating | 2.2 (5.1) | 2.1 (5.0) | 4.2 (10.4) | 8.2 (15.2) | 4.2 (10.7) | 2.7 (6.0) | 6.7 (9.1) | 5.7 (8.1) | 0.738 | *0.058* |
|  |  | Inactive | 9.4 (19.4) | 5.2 (10.4) | 13.1 (20.0) | 16.3 (22.4) | 13.8 (21.7) | 11.3 (22.0) | 10.4 (19.1) | 20.3 (25.3) | 0.510 | **0.014** |
| Values represent mean (standard deviation) unless otherwise noted. mg= milligram, VAS=visual analog scale | | | | | | | | | | | |  |

| Supplementary Table 2. Likert and Observed Time-based Analyses | | | | | | | | | | | |  |  |
| --- | --- | --- | --- | --- | --- | --- | --- | --- | --- | --- | --- | --- | --- |
|  |  |  | A118G AA | | | | A118G AG/GG | | | |  |  |  |
|  |  |  | Hydromorphone Dose | | | | Hydromorphone Dose | | | | Main Effects | | |
|  |  |  | 0mg | 2mg | 4mg | 8mg | 0mg | 2mg | 4mg | 8mg | A118G | Dose | Time |
| Patient-reported Likert Ratings (0-4) | | | | | | | | |  |  |  |  |  |
|  | Pleasant Ratings | | |  |  |  |  |  |  |  |  |  |  |
|  |  | Coasting | 0.0 (0.3) | 0.0 (0.2) | 0.1 (0.4) | 0.1 (0.3) | 0.6 (1.2) | 0.5 (1.1) | 0.5 (1.2) | 0.6 (1.2) | **<.0001** | 0.621 | **0.001** |
|  |  | Drive | 0.3 (0.8) | 0.3 (0.9) | 0.3 (0.9) | 0.3 (0.8) | 0.7 (1.5) | 0.7 (1.4) | 0.7 (1.4) | 0.8 (1.5) | **0.003** | 0.991 | **0.004** |
|  |  | Drunken | 0.0 (0.1) | 0.0 (0.0) | 0.0 (0.2) | 0.1 (0.4) | 0.0 (0.2) | 0.0 (0.0) | 0.0 (0.0) | 0.1 (0.5) | 0.728 | **<.0001** | **0.003** |
|  |  | Energetic | 0.6 (1.1) | 0.6 (1.1) | 0.7 (1.2) | 0.5 (1.0) | 1.0 (1.4) | 1.1 (1.6) | 1.2 (1.6) | 1.3 (1.6) | **0.000** | 0.883 | 0.819 |
|  |  | Flushing | 0.0 (0.1) | 0.0 (0.1) | 0.0 (0.2) | 0.0 (0.3) | 0.0 (0.0) | 0.0 (0.0) | 0.0 (0.2) | 0.1 (0.4) | 0.883 | **0.003** | **0.000** |
|  |  | Friendly | 1.3 (1.4) | 1.4 (1.4) | 1.3 (1.4) | 1.3 (1.4) | 1.5 (1.5) | 1.8 (1.6) | 1.7 (1.5) | 1.8 (1.6) | **0.028** | 0.734 | 0.163 |
|  |  | Pleasant Sick | 0.0 (0.1) | 0.0 (0.1) | 0.0 (0.2) | 0.0 (0.2) | 0.0 (0.0) | 0.0 (0.0) | 0.0 (0.1) | 0.1 (0.3) | 0.903 | **0.019** | 0.252 |
|  | Unpleasant Ratings | |  |  |  |  |  |  |  |  |  |  |  |
|  |  | Blurred Vision | 0.0 (0.0) | 0.0 (0.0) | 0.0 (0.1) | 0.0 (0.2) | 0.0 (0.0) | 0.0 (0.1) | 0.0 (0.0) | 0.0 (0.2) | 0.502 | **0.008** | 0.232 |
|  |  | Feel Limp or Loose | 0.1 (0.5) | 0.0 (0.2) | 0.2 (1.2) | 0.2 (0.6) | 0.0 (0.0) | 0.0 (0.1) | 0.0 (0.2) | 0.2 (0.6) | 0.129 | **0.009** | 0.873 |
|  |  | Headache | 0.1 (0.3) | 0.1 (0.3) | 0.1 (0.4) | 0.2 (0.5) | 0.1 (0.4) | 0.2 (0.4) | 0.2 (0.5) | 0.1 (0.3) | 0.191 | 0.380 | **0.009** |
|  |  | Mentally Slowed Down | 0.2 (1.4) | 0.1 (0.5) | 0.4 (1.6) | 0.3 (0.9) | 0.1 (0.3) | 0.1 (0.4) | 0.1 (0.3) | 0.3 (0.6) | 0.078 | **0.020** | 0.288 |
|  |  | Nervous | 0.0 (0.2) | 0.0 (0.2) | 0.0 (0.3) | 0.0 (0.2) | 0.1 (0.3) | 0.1 (0.2) | 0.1 (0.3) | 0.1 (0.4) | **0.047** | 0.730 | **0.020** |
|  |  | Sick to Stomach | 0.0 (0.2) | 0.0 (0.2) | 0.2 (0.6) | 0.2 (0.5) | 0.0 (0.0) | 0.0 (0.0) | 0.0 (0.2) | 0.1 (0.3) | **0.012** | **0.005** | 0.640 |
|  |  | Skin Itchy | 0.0 (0.3) | 0.0 (0.3) | 0.1 (0.3) | 0.2 (0.6) | 0.0 (0.3) | 0.0 (0.3) | 0.0 (0.2) | 0.0 (0.2) | **0.031** | 0.053 | 0.188 |
|  |  | Turning of Stomach | 0.1 (0.3) | 0.1 (0.3) | 0.2 (0.5) | 0.2 (0.5) | 0.0 (0.0) | 0.0 (0.1) | 0.0 (0.2) | 0.1 (0.3) | **0.004** | **0.045** | 0.534 |
| Observed VAS Ratings (0-100) | | | | | | |  |  |  |  |  |  |  |
|  | Nonspecific Ratings | | | | |  |  |  |  |  |  |  |  |
|  |  | Drug Effects | 2.35 (9.2) | 1.69 (5.58) | 7.12 (14.14) | 7.7 (14.82) | 1.73 (5.2) | 0.73 (3.61) | 4.23 (10.9) | 9.8 (16.99) | 0.461 | **<.0001** | **<.0001** |
|  |  | Good Effects | 0.68 (4.55) | 0.31 (2.84) | 0.94 (5.47) | 1.16 (5.89) | 0.1 (1.37) | 0 (0) | 0.11 (0.81) | 1.41 (8.39) | 0.376 | 0.200 | 0.206 |
|  |  | Bad Effects | 0.97 (6.03) | 0.64 (3.61) | 3.13 (9.15) | 3.93 (10.86) | 0.47 (3.45) | 0 (0) | 0.47 (2.27) | 2.66 (8.76) | **0.039** | **0.007** | **0.030** |
|  |  | High | 0.39 (4.16) | 0.18 (1.23) | 1.58 (7.32) | 2.02 (8.67) | 0 (0) | 0 (0) | 0.92 (4.55) | 2.03 (10.06) | 0.523 | **0.047** | 0.076 |
|  | Pleasant Ratings | | |  |  |  |  |  |  |  |  |  |  |
|  |  | Energized | 1.67 (8.22) | 1.36 (7.58) | 0.71 (3.12) | 1.22 (5.91) | 0.27 (1.6) | 0.63 (7.33) | 0.41 (2.95) | 1.11 (5.19) | 0.202 | 0.834 | 0.643 |
|  |  | Talkative | 7.35 (15.88) | 8.26 (17.27) | 7.25 (14.77) | 8.72 (16.87) | 13.6 (18.06) | 14 (19.87) | 13.76 (19.98) | 16.96 (22.35) | **<.0001** | 0.449 | **0.000** |
|  |  | Stimulated | 1.87 (10.88) | 1.23 (7.79) | 0.79 (6.43) | 1.12 (7.45) | 0.87 (5.99) | 1.57 (11.7) | 1.25 (9.69) | 1.26 (4.74) | 0.854 | 0.909 | 0.423 |
|  | Unpleasant Ratings | | | | |  |  |  |  |  |  |  |  |
|  |  | Sedated | 0.82 (6.14) | 0.58 (3.17) | 1.59 (6.98) | 1.85 (8.67) | 0.76 (3.61) | 0.21 (1.58) | 0.67 (3.21) | 1.08 (4.9) | 0.326 | 0.552 | **0.025** |
|  |  | Sleepy | 2.65 (10.03) | 3.44 (10.81) | 7.08 (15.61) | 8.24 (17.44) | 4.87 (12.17) | 3.02 (10.48) | 2.28 (8.2) | 7.73 (16.05) | 0.346 | **0.003** | **0.003** |
|  |  | Difficulty Concentrating | 0.46 (4.72) | 0.41 (3.55) | 0.84 (4.84) | 1.41 (6.29) | 0.66 (3.49) | 0.29 (1.88) | 1.07 (3.49) | 1.05 (3.75) | 0.895 | 0.097 | **0.009** |
|  |  | Inactive | 2.24 (9.53) | 1.25 (6.7) | 3.18 (10.43) | 4.51 (13) | 3.81 (13.07) | 2.2 (7.74) | 2.58 (8.05) | 6.9 (15.09) | 0.177 | **0.001** | **<.0001** |
| Values represent mean (standard deviation) unless otherwise noted. mg= milligram, VAS=visual analog scale | | | | | | | | | | | | |  |
